# Pigment epithelium detachment with thickened choroid in a preterm infant at term-equivalent age: a case report

**DOI:** 10.3389/fmed.2025.1581191

**Published:** 2025-08-12

**Authors:** Seong Joon Ahn, Vincent Tai, Katrina P. Winter, Neeru Sarin, Cynthia A. Toth

**Affiliations:** ^1^Department of Ophthalmology, Duke University School of Medicine, Durham, NC, United States; ^2^Department of Ophthalmology, Hanyang University Hospital, Hanyang University College of Medicine, Seoul, Republic of Korea

**Keywords:** optical coherence tomography, choroid, pigment epithelium detachment, preterm, steroid

## Abstract

Pigment epithelial detachment (PED) is well-documented in adult retinal diseases but is rarely reported in neonates. This case describes a preterm infant, born at 31 weeks, who developed PED with thickened choroid at term-equivalent age, detected using handheld OCT. The PED emerged at 39 weeks postmenstrual age, coinciding with inhaled steroid treatment for respiratory distress, and resolved by 41 weeks after steroid discontinuation without structural damage. The temporal relationship suggests an association between steroid use and PED with choroidal changes. This case highlights the value of OCT in detecting retinal and choroidal abnormalities in preterm infants and underscores the potential impact of systemic conditions and medications on the choroid.

## 1 Introduction

Pigment epithelial detachment (PED) is characterized by the separation of the retinal pigment epithelium (RPE) from the underlying Bruch’s membrane most commonly due to the accumulation of fluid in the sub-RPE space (1). PED is commonly associated with retinal disorders such as pachychoroid pigment epitheliopathy and central serous chorioretinopathy (2–4) and can serve as critical markers for disease severity and progression (2, 5). PEDs are primarily identified using optical coherence tomography (OCT) as dome-shaped elevations of the RPE (6).

PED, typically considered an age-related feature, has rarely been reported in infants. One report described presumed bilateral multiple PED in a preterm infant based on fundus photography (7); however, it lacked OCT confirmation and detailed clinical information to assess any association with systemic conditions. This rarity is likely due to the limited availability of imaging techniques capable of detecting subtle retinal changes in this population. However, the advent of handheld OCT has revolutionized retinal imaging in infants, allowing the identification of features previously observed primarily in adults, such as macular edema, retinoschisis, and abnormal retinal vasculature, including preretinal neovascularization (8). These advancements provide new insights into the pathological and developmental aspects of the infant retina that were previously less understood.

This report presents the case of a preterm infant born at 31 weeks of gestation who exhibited PED and choroidal thickening at a term-equivalent age on handheld OCT. This case contributes to the limited literature on PED in neonates and provides new insights into PED incidence and its association with choroidal thickening in this population.

## 2 Case description

A female preterm infant of African descent was born at 31 + 1 weeks of gestation, with a birth weight of 840 g. The pregnancy was complicated by severe preeclampsia and oligohydramnios, and the mother received maternal steroids. At birth, the infant had APGAR scores of 1 and 3 at 1 and 5 min, respectively, indicating significant neonatal distress. Her postnatal course was further complicated by clinical sepsis, pulmonary edema, adrenal insufficiency, patent foramen ovale, mitral regurgitation with a moderate atrial septal defect, and electrolyte abnormalities (Table 1).

**Table 1:** Clinical summary and ocular findings.

PMA	Event/Intervention	Ocular findings
31 + 1 weeks	Birth: 840 g SGA female; intubation & surfactant; clinical sepsis; pulmonary edema; adrenal insufficiency; PFO; mitral regurgitation; ASD; electrolyte abnormalities	
37 + 0 weeks	Baseline handheld OCT (no steroids yet)	Grade 1 ROP in both eyes; OCT: macular edema with cystoid spaces in the inner nuclear layer; Choroidal thickness: 298.7 μm in the right eye, 316.0 μm in the left
38 + 0 to 39 + 0 weeks	Budesonide (Pulmicort®) 0.5 mg BID via face-mask nebulizer for 7 days (14 doses; 7 mg cumulative)	
39 + 0 weeks	OCT at end of steroid course	ROP stable at Grade 1; OCT: dome-shaped subfoveal PED in left eye; Choroidal thickness: increased to 329.0 μm in the right and 368.0 μm in the left
41 + 0 weeks	Follow-up handheld OCT	ROP stable OCT: persistent macular edema; resolution of PED Choroidal thickness returned to 303.0 μm in the right and 307.4 μm in the left

The infant was intubated shortly after birth and required surfactant therapy. Pulmonary edema persisted throughout hospital stay, necessitating the administration of diuretics. Budesonide (Pulmicort, AstraZeneca, Cambridge, UK) was administered via inhalation between 38 and 39 weeks of postmenstrual age (PMA) to manage respiratory distress and associated tachypnea. Inhaled budesonide (Pulmicort®) was delivered via face-mask jet nebulizer at 0.5 mg twice daily for seven days (14 doses; 7 mg cumulative). At 41 weeks PMA, she continued to exhibit intermittent tachypnea but remained stable in normal room air.

**Figure 1:**
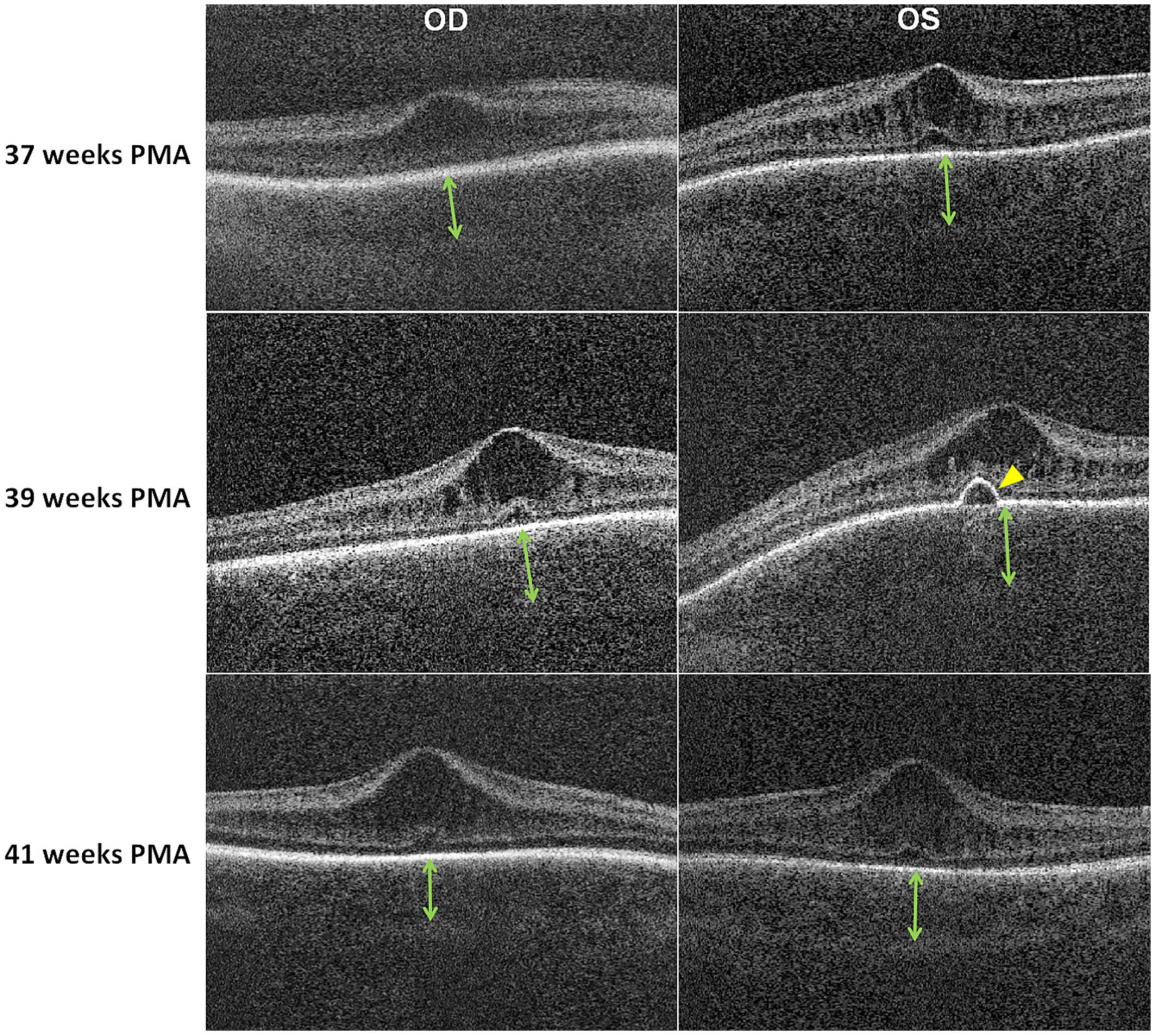
Serial OCT images obtained at 37, 39, and 41 weeks postmenstrual age (PMA) in an infant born at 31 weeks gestation. At 39 weeks PMA, 1 week after steroid inhalation, choroidal thickening was observed in both eyes (indicated by arrows) compared to the eyes on the 37-week visit. The left eye at 39 weeks PMA showed pigment epithelium detachment, visible as bulging of the retinal pigment epithelium and Bruch’s membrane complex (yellow arrowhead), which resolved by 41 weeks PMA, following 1 week of discontinuation of steroid inhalation. By 41 weeks PMA, the choroidal thickness had decreased to levels similar to those observed at 37 weeks PMA in both eyes.

Grade 1 retinopathy of prematurity (ROP) was observed during routine ophthalmologic screening, conducted with pupil dilation using Cyclomydril eyedrops (cyclopentolate hydrochloride 0.2%, phenylephrine hydrochloride 1%; Alcon Laboratories, Fort Worth, TX). No signs of additional disease or progression were noted, and the anterior segment and vitreous humor were unremarkable. OCT images were obtained at 37 weeks PMA using an investigational portable handheld swept-source device (8) with fovea-centered horizontal line scans and showed macular edema characterized by cystoid spaces in the inner nuclear layer typical of those seen in preterm infants (9) with no notable RPE abnormalities or choroidal features (Figure 1). However, at 39 weeks PMA, corresponding to the last day of steroid inhalation, OCT images were acquired using horizontal line scans centered on the anatomical subfovea across all follow-up visits, revealing a dome-shaped PED in the subfoveal area of the left eye (Figure 1, arrows), accompanied by a change in the choroidal inner boundary contour from nearly flat to convex. The choroid was thickened in both eyes, with an indistinct choroid-scleral junction compared with the previous visit. Choroidal thickness, measured using DOCTRAP (10) by averaging over the central 2 mm across the fovea on horizontal line scans, was 298.7 μm in the right eye and 316.0 μm in the left eye at 37 weeks PMA. By 39 weeks PMA, the thickness had increased to 329.0 μm in the right eye and 368.0 μm in the left eye.

At the follow-up visit at 41 weeks PMA, macular edema persisted in both eyes; however, the PED resolved without structural changes in the RPE/Bruch’s membrane complex. The choroid thinned to 303.0 μm in the right eye and 307.4 μm in the left eye, returning to levels similar to those observed at 37 weeks PMA.

## 3 Discussion

This report describes a case of thickened choroid with transient PED in a preterm infant, confirmed by OCT. A previous report on a presumed PED in a preterm infant, identified through fundus photography, attributed the cause to the effect of 2.5% phenylephrine on choroidal circulation and the RPE (7). In our case, 1% phenylephrine was used for pupil dilation at all three visits, making it unlikely to explain the PED observed only at the second visit. Although the etiology of PED in preterm infants remains unclear, potential contributing factors include increased hydrostatic pressure in the choroidal circulation, which can be associated with certain systemic conditions and medications (11, 12). Notably, the close temporal association between steroid use and the occurrence and disappearance of PED in our case suggests a link among steroid use, choroidal thickening, PED development, and lack of pigmentary change.

Specifically, serial OCT images showed a temporal correlation between steroid use, presence of PED, and choroidal thickening. Choroidal changes in preterm infants was previously documented, with earlier studies depicting choroidal thickness as independently associated with several systemic conditions, such as impaired baseline health metrics, cardiac and pulmonary abnormalities, slower postnatal growth velocity, and the need for supplemental oxygen (13). Systemic insults—sepsis-driven inflammation, respiratory distress with fluctuating oxygenation, and high-output cardiac shunts—can increase choroidal vascular permeability, alter blood-flow dynamics, and raise hydrostatic pressure, thereby promoting choroidal thickening and PED formation. In addition, systemic steroid use increases choroidal hyperpermeability, causes choroidal thickening (12, 14). As the PED and choroidal thickening observed in our patient is consistent with prior observations from steroid users across a broad age range (11, 12, 14), the initiation of steroid inhalation 1 week pre-PED occurrence likely played an important role in its pathogenesis.

PED disappearance upon steroid cessation highlights the potential reversibility of these changes in the neonatal population as well as a stronger temporal relationship between steroid use (discontinuation) and our findings. In our case, there was no remarkable abnormality in the RPE following PED resolution, suggesting no sequelae on the RPE, which should be proven with long-term follow-up imaging. Additionally, this emphasizes the role of OCT imaging in the detection and follow-up of PED in preterm infants receiving steroid treatment for systemic conditions such as bronchopulmonary dysplasia, and the importance of reviewing systemic care when considering the cause of ocular conditions in infants.

However, the single-case design limits generalizability, so larger series or cohort studies are needed. The link between budesonide and PED is based on timing and is not definitive. Short follow-up prevents assessment of long-term outcomes—extended OCT monitoring would help. Finally, other factors like systemic inflammation, blood pressure changes, or diuretic use could have affected choroidal thickness.

## 4 Conclusion

This report presents an OCT-documented case of PED with choroidal thickening in a preterm infant at term-equivalent age. To the best of our knowledge, we could not find any previously reported cases in PubMed and Embase (January 2000–February 2025) with similar findings. The temporal association between steroid use/discontinuation and PED development/disappearance suggests that steroid treatment may play a role in choroidal and retinal pathologies, even at this young age. Further studies including larger cohorts and longitudinal research are essential to understanding the prevalence, clinical significance, and natural history of PED in preterm infants. In addition, choroidal changes observed on OCT may serve as biomarkers of systemic conditions and medication effects, offering a novel avenue for exploring the interplay between systemic health or medication use, and retinal/choroidal pathologic features in the neonatal population.

## Data Availability

The original contributions presented in the study are included in the article/supplementary material, further inquiries can be directed to the corresponding author/s.
